# The Research Progress in Physiological and Pathological Functions of TRAF4

**DOI:** 10.3389/fonc.2022.842072

**Published:** 2022-02-15

**Authors:** Xueqin Ruan, Rong Zhang, Ruijuan Li, Hongkai Zhu, Zhihua Wang, Canfei Wang, Zhao Cheng, Hongling Peng

**Affiliations:** ^1^ Department of Hematology, The Second Xiangya Hospital, Central South University, Changsha, China; ^2^ Institute of Molecular Hematology, Central South University, Changsha, China; ^3^ Division of Cancer Immunotherapy, National Cancer Center Exploratory Oncology Research & Clinical Trial Center, Chiba, Japan; ^4^ Hunan Key Laboratory of Tumor Models and Individualized Medicine, The Second Xiangya Hospital, Central South University, Changsha, China

**Keywords:** TRAF4, carcinogenesis, apoptosis, proliferation, pathological

## Abstract

Tumour necrosis factor receptor-associated factor 4 (TRAF4) is a member of the TRAF protein family, a cytoplasmic bridging molecule closely associated with various immune functions. The physiological processes of TRAF4 are mainly involved in embryonic development, cell polarity, cell proliferation, apoptosis, regulation of reactive oxygen species production. TRAF4 is overexpressed in a variety of tumors and regulates the formation and development of a variety of tumors. In this review, we summarize the physiological and pathological regulatory functions of TRAF4 and focus on understanding the biological processes involved in this gene, to provide a reference for further studies on the role of this gene in tumorigenesis and development.

## 1 TRAF Family

Tumour necrosis factor receptor (TNFR)-associated factor (TRAF) is a critical linker molecule in the tumor necrosis factor superfamily (TNFSF) and Toll-like/interleukin-1 receptor (TLR/ILR) superfamily that plays a regulatory role in cell proliferation, differentiation, apoptosis and survival, and immune response. TRAF family members all have TRAF homologous structural domains at the carboxyl terminus, zinc-finger of the amino terminus, and ring finger structural domains. TRAF is a cytoplasmic bridging protein class that binds to other TRAF proteins after connecting to the receptor’s cytoplasmic domain. Six classical members (TRAF1-TRAF6) and one non-classical member (TRAF7) are known in mammals. TRAF2-6 has ring and zinc-finger motifs that are important for the downstream regulation of signaling (1). Structural features of TRAF proteins suggest that these proteins function as cytoplasmic junctions that promote intracellular signaling through their ability to bind to receptors and enhance the recruitment of proteins to signaling complexes (2).

In terms of structure, the TRAF-N region of the TRAF family is typically composed of more than ten 7-peptide repeats. In contrast, this region of TRAF4 contains only three 7-peptide repeats and the relatively short coiled helix structural domain of TRAF4 results in weak binding of the TRAF4 protein to other TRAF family proteins. Although the TRAF domain of TRAF4 has a typical TRAF domain fold, its high-resolution structure reveals similarities and differences between this structure and other TRAF family members, and these structural differences lead to functional variability (3). TRAF4 is the only TRAF family member with three CART structural domains, for which TRAF4 was once named CART1. Only TRAF4 in the TRAF family contains a unique N-terminal RING-finger and a nuclear localization signal (NLS), and TRAF4 may belong to a subfamily of TRAFs that function in the nucleus (4).

In terms of origin, the TRAF1, 2, 3 and 5 genes arose from the duplication of recent independent genes and had a common ancestor gene. Correlative evolutionary analyses show that TRAF1, 2, 3 and 5 emerged during vertebrate evolution. The TNFR family was formed at this same stage, suggesting that the functions of these four TRAF family proteins are related to those of the TNFR family proteins. Furthermore, TRAF1, 2, 3, 5 and 6 have been shown to interact directly or indirectly with the TNFR superfamily (5, 6). TRAF4 emerged earlier in evolution, homologous analogues of TRAF4 have been identified in lower coelenterates, and TRAF4 homologues play similar roles during embryonic development in both invertebrates and vertebrates, leading to the inference that TRAF4 may be one of the older members of the TRAF family and that it exerts a function that is TNFR non-dependent (7).

In terms of function, unlike other members of the TRAF protein family, TRAF4 migrates in the cell membrane, cell plasma and nucleus mainly through indirect action or formation of complexes, thus participating in several signalling pathways such as NF-κB and JNK to exert physiopathological regulatory functions. However, TRAF4 is less capable of activating signalling pathways alone and must be combined with other interacting proteins before it can effectively activate signalling pathways (8, 9). 

## 2 Physiological Regulation Function of TRAF4

Tumour necrosis factor (TNF) receptor-associated factor 4 (TRAF4) is an E3 ubiquitin ligase and a member of the tumor necrosis factor receptor-associated factor (TRAF) family, which is a crucial molecule in individual development. TRAF4 contains a nuclear localization signal (NLS), a unique TRAF family (4). The TRAF4 amino-terminal ring finger (RING) domain has E3 ubiquitin ligase activity, which promotes the ubiquitination of related proteins, regulates downstream signaling (10), and mediates activation of TAK1 and AKT1 target proteins and ubiquitination of K63 linkages (11). TRAF4 is involved in the transduction of multiple signaling pathways, and most of the biological effects of TRAF4 accompany the activation of signaling pathways activation (**Table 1**).

**Table 1:** Physiological regulation of TRAF4.

Regulating factor	Signaling pathway	Function	Impact	PMID
Rock2 (RhoA**’**s Downstream target)	NgR/p75NTR/RhoA	Reduce actin cytoskeleton rearrangement	Reduce axonal regeneration inhibition and neuronal apoptosis	22363515
Smurf1	BMP and Nodal signaling	Involved in neural crest development and neural plate morphogenesis	Affects nerve formation	19458200
TGF-β	Tight junction-dependent signaling pathway	Epithelial-mesenchymal translocation and p53 instability	Affects cell polarity	25610759
EGFR (Epidermal growth factor receptor)	EGFR autophosphorylation and downstream signaling	Induction of conformational rearrangements in the JM region	Induction of cell proliferation, migration, and differentiation	30352854
TNFR (GITR)	NF-κB pathway	Regulation of the suppressive function of Treg cells	Promotes the activation of T cells involved in immune function	15583869
miR-4443	TRAF4/Iκα/NF-κB signaling pathway	Key media for SIDS	Activation of anti-inflammatory cytokine expression in monocytes	32337817
miR-4443	NF-κB pathway	Increased secretion and proliferation of CD4+ T-cell cytokines	Results in Graves’ Disease	29163513
NOD2	NF-κB pathway	Induced bacterial killing	Causes Crohn’s disease	22547678
SMURF2 (E3-ligase)	DAZAP2 degradation	Initiate IL-25 signal	Causes airway inflammation	25681341
Beclin-1	Inhibition of Beclin-1 phosphorylation	Impairment of lipopolysaccharide-induced apoptotic autophagy	Induction ankylosing spondylitis	28604663
NTR(Dimeric neurotrophic factor receptor p75	Reduced dimerization	Inhibition of NF activation response	Induction of apoptosis	10514511
STEAP4	IL-17/TNF-α-TRAF4-STEAP4 axis	Regulating copper homeostasis	Induced copper-mediated oxidative damage	33179273
Atox1 (Copper-dependent antioxidant-1)	Stimulation of TNF-α	Nuclear translocation	Induces a reactive oxygen-dependent inflammatory response	31553645
Rho GTPases and p21-activated kinase 1 (PAK1)	PTP-PEST focal contact phosphatase	Starter film wrinkling	Involvement in Oxidative modifications	16330715
ALKBH5	N6-methyladenosine RNA demethylation	Regulation of adipogenesis in MSCs	Fine tuning of lipo-osteogenic differentiation balance	32268273
Smurf2	ubiquitination	Positive regulation of osteogenic differentiation of MSCs	Causes disorders of bone metabolism	31076633
MEKK4	Co-activation of JNK	Fine-tuning the assembly of mesodermal cell adhesion junctions	Impact repair function	16157600
USP1 (Deubiquitinating enzyme)	Inhibition of p53 pathway	Causes p53 instability	Regulation of proliferation in induced skin repair	30940456
UPS	TGF-β/Smad signaling pathway	Causes p53 instability	Causes Development and progression of hypertrophic scars	33433895

## 3 Non-Tumor Function of TRAF4

**Figure 1:**
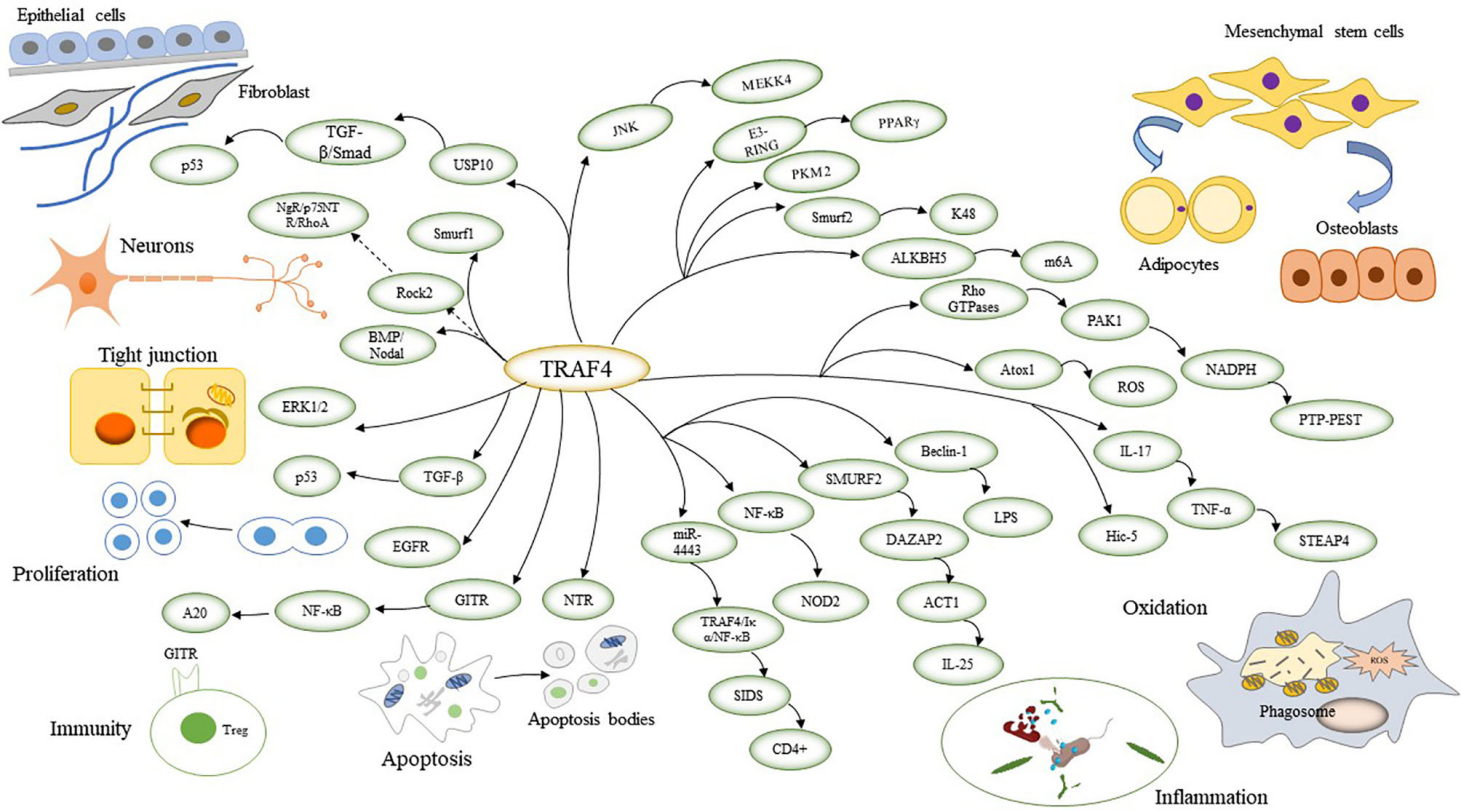
TRAF4 involves signalling pathways that link physiological functions by connecting to reciprocal genes. TRAF4 regulates neuronal cell formation and affects cell polarity by regulating tight junctions. TRAF4 also promotes cell proliferation and cell inflammation and inhibits apoptosis. TRAF4 affects cellular oxidation by inducing ROS. TRAF4 regulates the differentiation of mesenchymal stem cells into adipocytes and osteoblasts, thereby impacting adipose bone production and metabolism. TRAF4 promotes cellular repair by affecting fibroblasts.

TRAF4 is required during embryogenesis and is mainly involved in neurogenesis, cell polarity, cell proliferation, immunity, inflammation-related functions, apoptosis, oxidation, osteogenesis metabolism, and repair (12) (**Figure 1**).

In neurogenesis, in the absence of TRAF4, increased phosphorylation of Rock2 (a downstream target of RhoA) leads to activation of the NgR/p75NTR/RhoA signaling pathway induces actin cytoskeletal rearrangement and facilitates axonal regeneration inhibition and neuronal apoptosis (13). TRAF4 is identified as a novel target of Smurf1 that polyubiquitinates TRAF4 to trigger its proteasomal destruction. TRAF4 enhances bone morphogenetic proteins BMP and Nodal signaling. TRAF4 functions as a novel Smurf1 regulatory mediator of BMP and Nodal signaling, essential for neural crest development and neural plate morphogenesis (14).

In cell polarity, TRAF4 is highly mobile and shuttles between the tight cell junction (TJ) and the cytoplasm. Intracellular TRAF4 enhances ERK1/2 phosphorylation in proliferating HeLa cells, an epithelial cell line that lacks TJ. TRAF4 is involved in an emerging TJ-dependent signaling pathway that affects cell polarity by regulating cell proliferation/differentiation homeostasis and subsequent epithelial homeostasis (15). TRAF4 contributes to TGF-β-induced epithelial-mesenchymal transition (EMT), metastasis, and p53 instability (16).

In cell proliferation, activation of the epidermal growth factor receptor (EGFR) induces changes in cellular functions such as proliferation, migration, and differentiation. TRAF4 binding induces conformational rearrangements in the JM region to promote EGFR dimerization, and TRAF4 overexpression promotes EGF-induced EGFR autophosphorylation and downstream signaling. In contrast, deletion of TRAF4 binding sites and specific point mutations significantly attenuated EGFR activation and EGF-driven cell proliferation (17).

In immunity, TRAF4 is involved in immune function by promoting immune cell migration (18). TRAF4 enhances glucocorticoid-induced TNFR (GITR), a receptor expressed on T cells, B cells, and macrophages, thereby triggering activation of NF-kappaB. TRAF4-mediated activation of NF-kappaB downstream of GITR depends on the TRAF binding site in the previously mapped receptor cytoplasmic domain and is inhibited by cytoplasmic protein A20. TRAF4 is thought to inhibit the suppressive function of regulatory T cells (Treg cells) and promote T cell activation. TRAF4 elaborates on GITR signaling and has been shown to regulate the suppressive function of Treg cells (19). TRAF4 binding motifs are involved in the termination of innate immune signaling responses (20).

In terms of inflammation-related functions, miR-4443 overexpression inhibited the TRAF4/Iκα/NF-κB signaling pathway to activate anti-inflammatory cytokine expression in monocytes. miR-4443 increased expression-induced monocyte dysfunction by targeting TRAF4, which may be a key mediator of SIDS (21). MiR-4443 directly inhibited TRAF 4 expression, increasing CD4+ T cell cytokine secretion and proliferation *via* the NF-κB pathway. Increased miR-4443 expression induces CD4+ T cell dysfunction by targeting TRAF4, leading to GD (22). TRAF4 is a critical negative regulator of NOD2 signaling. TRAF4 may be involved in regulating and coordinating the immune response to the intracellular pattern recognition receptor NOD2, which negatively regulates NOD2 through two consecutive glutamate residues of NOD2, leading to NF-κB pathway activation. TRAF4 inhibits NOD2-induced NF-κB activation and binds directly to NOD2 to inhibit NOD2-induced bacterial killing. TRAF4 phosphorylation and subsequent inhibition of NOD2 signaling require binding to the Crohn’s disease susceptibility protein NOD2 (23).

TRAF4 deficiency leads to tracheal malformations, resulting in altered airflow and increased lung inflammation (24). TRAF4 recruits the E3-ligase SMURF2 to degrade the IL-25R inhibitory molecule DAZAP2. TRAF4-SMURF2-mediated degradation of DAZAP2 is a crucial component of ACT1 recruitment to IL-25R to initiate IL-25 signaling and a critical step in airway inflammation (25). Increased TRAF4 expression in ankylosing spondylitis mesenchymal stem cells (MSCs) may impair lipopolysaccharide (LPS)-induced apoptotic autophagy by inhibiting Beclin-1 phosphorylation (26).

In terms of apoptosis, TRAF4 interacts with the dimeric neurotrophic factor receptor p75(NTR), and TRAF4 overexpression reduces its ability to dimerize to prevent the induction of apoptosis mediated by monomeric p75(NTR). TRAF4 also inhibits the NF activation response (27). TRAF4 is localized in the cytoplasm and appears to be retained in the cytoplasm after DNA damage Overexpression of TRAF4 induces apoptosis and inhibits colony formation (28).

In terms of oxidation, IL-17 and TNF-α induce upregulation of STEAP4 largely dependent on changes in TRAF4. TRAF4 is resistant to copper-mediated oxidative damage. The IL-17/TNF-α-TRAF4-STEAP4 axis can be used to regulate copper homeostasis (29). In TNF-α-stimulated endothelial cells (ECs), the binding of copper-dependent antioxidant-1 (Atox1) to TRAF4 leads to Atox1 nuclear translocation and induces a reactive oxygen species (ROS)-dependent inflammatory response (30). TRAF4 is directly associated with focal contact scaffolding (Hic-5), and knockdown of the protein, disruption of the complex, or oxidant scavenging prevents cell migration. Active mutants of TRAF4 activate Rho GTPases and p21-activated kinase 1 (PAK1) downstream of NADPH oxidase and oxidatively modify focal contact phosphatase (PTP-PEST). TRAF4 initiates robust membrane puckering *via* Rac1, PAK1, and oxidase, whereas knockdown of PTP-PEST increases membrane puckering while being unrelated to oxidase activation unrelated. TRAF4 specifies the molecular address in the focal complex targeting oxidative modifications during cell migration (31).

In terms of osteogenic metabolism, TRAF4 negatively regulates adipogenesis in MSCs through activation of PKM2 kinase activity, and downregulation of TRAF4 during adipogenesis is regulated by ALKBH5-mediated N6-methyladenosine RNA demethylation. This may serve as a checkpoint for fine-tuning the balance of adipose-osteogenic differentiation and suggests that TRAF4 may be a novel target for clinical MSCs and may also elucidate potential mechanisms of bone metabolic diseases (32). TRAF4 positively regulates osteogenesis in MSCs both *in vitro* and *in vivo*. TRAF4 acts as an E3 ubiquitin ligase that degrades Smurf2 to regulate the osteogenic differentiation of MSCs positively. TRAF4 plays a crucial role in bone formation to elucidate the pathogenesis of bone metabolism disorders and improve the practical clinical application of MSCs (33). TRAF4 acts as an E3-RING ubiquitin ligase, promotes the degradation of the adipocyte differentiation regulator PPARγ through the ubiquitin-proteasome pathway, thereby inhibiting adipogenesis.

In terms of repair, TRAF4 acts in a TNF receptor- and JNK-independent manner to fine-tune the assembly of adhesion junctions in invaginated mesangial cells (34). TRAF4 stimulates MAPK/ERK kinase 4 (MEKK4) activity by promoting MEKK4 oligomerization, and JNK activation can be facilitated by chemically inducing MEKK4 dimerization. MEKK4 is a TRAF4-regulated JNK pathway of MAPK kinase (9). TRAF4 inhibits the p53 pathway independent of its E3 ubiquitin ligase activity. TRAF4 interacts with the deubiquitinating enzyme USP10 and blocks p53 from entering USP10, resulting in p53 instability. TRAF4 proliferation regulation in skin repair is dependent on p53 (35). UPS is involved in skin repair by regulating the transforming growth factor (TGF)- β/Smad signaling pathway in the development and progression of hypertrophic scars. TRAF4 binding to the deubiquitinating enzyme USP10 induces p53 instability and promotes scar tissue-derived fibroblasts and skin repair (36). 

## 4 Role of TRAF4 in Breast Cancer

### 4.1 Migration Regulatory Role in Breast Cancer

TRAF4 expression is suppressed in the nucleus of breast cancer cells and is associated with the invasive ability of breast cancer (37). TRAF4, known initially as CART1, is specifically expressed in breast cancer and localized in the nucleus in such tissues. Breast cancer cell proliferation is dependent mainly on TRAF4, and overexpression of TRAF4 can contribute to breast cancer progression by destabilizing the tight junctions (TJs) between cells, thereby promoting cell migration (38). TRAF4 recruits the TJs and prevents their formation and stabilization, facilitating cell migration that allows cancer progression. TRAF4 is dynamically localized to the TJs and is overexpressed in cancers. TRAF4 is a protein dynamically localized to the TJ and overexpressed in cancer and plays multiple functions in breast cancer progression (39). Girdin is mainly expressed in the cytoplasm of breast cancer cells, and TRAF4 facilitates its translocation to the nucleus. Cytoplasmic expression of TRAF4 may be a new potential marker of breast cancer cell migration (40).

### 4.2 Proliferation Regulatory Role in Breast Cancer

TRAF4 upregulates PRMT5 expression in the nucleus, and PRMT5 forms a specific zinc finger structure with TRAF4, which plays an essential role in activating the NF-κB signaling pathway and promotes breast cancer cell proliferation (41). Overexpression of TRAF2 enhances the cytoplasmic expression of TRAF4, which promotes cell proliferation and suppresses NF-κB nuclear transcription through activation of apoptosis (42). Reducing TRAF4 inhibits proliferation, invasion, and metastasis of breast cancer cells by downregulating the AKT signaling pathway, inactivating the NF-κB path, and engaging the interaction of RSK4 (43). TRAF4 promotes membrane recruitment of the cell survival kinase AKT. The overexpression of activated AKT reverses cell growth arrest in TRAF4-silenced cells, which plays an essential role in breast carcinogenesis by activating and interacting with AKT. Overexpression of TRAF4 enhances IGF1-induced IGFR-IRS-1 interaction, IRS-1 tyrosine phosphorylation, and activation of AKT and ERK effector proteins downstream of IGF-1, while mutation of the IRS-1 ubiquitination site eliminates these effects (44).

### 4.3 Anti-Apoptotic Regulatory Role in Breast Cancer

Reducing TRAF4 increases the proportion of early to mid-stage apoptotic cells in MCF-7 cells, growing G1-phase cells, and decreasing S-phase cells detected. TRAF4 has an anti-apoptotic effect on TNF-α-induced apoptosis in MCF-7 cells (45). By promoting the activation of p70s6k signaling through upregulation of cytoplasmic expression of TRAF4, the p70s6k/S6 signaling pathway plays an essential role in promoting cell proliferation TRAF4 (46). Expression of SRC-3 was inversely correlated with the expression of p53-regulated proapoptotic genes in breast cancer, and SRC-3 and TRAF4 overexpression reduced cytotoxic stress-induced tumor suppression factor p53 protein upregulation. Breast cancer cells overexpressing TRAF4 were more resistant to stress-induced death. TRAF4-mediated inhibition of herpesvirus-associated ubiquitin-specific protease (HAUSP) subsequently led to the loss of p53 deubiquitination and its stability in the cellular stress response. TRAF4 overexpression in breast cancer patients was significantly associated with poor prognosis (47). TRAF4 was identified as a p53 target gene, and TRAF4 overexpression inhibited colony formation (48). TRAF4 is essential for migrating normal breast epithelial cells and breast cancer cells. The ability of TRAF4 to promote cell migration also depends on Smurf1-mediated ubiquitination, which is associated with TRAF4 activation of Rac1 (49).

### 4.4 Drug Resistance Regulatory Role in Breast Cancer

The β-catenin was identified as a TRAF4-binding protein, TRAF4 enhances β-catenin expression, and TRAF4 mediates the translocation of β-catenin from the cytoplasm the nucleus, thereby facilitating the Wnt signaling pathway in breast cancer (8). TRAF4 is a novel substrate for SIAH1 and prevents SIAH1-mediated β-catenin degradation, the protective effect of TRAF4 on β-catenin during cellular stress, and linking TRAF4 to tumor chemoresistance, TRAF4 reduces the growth inhibitory effects of chemotherapeutic agents such as etoposide by reducing the number of S-phase cells and inhibiting apoptosis (50). TRAF4 is a crucial component that mediates pro-oncogenic transforming growth factor-β (TGF-β)-induced SMAD and non-SMAD signaling. TRAF4 is required for effective TGF-β-induced migration, epithelial-mesenchymal transition, and breast cancer metastasis. In breast cancer patients, elevated TRAF4 expression is associated with increased phosphorylated SMAD2 and phosphorylated TAK1 and poor prognosis. TRAF4 regulates the TGF-β pathway, promotes TGF-β receptor signaling, and drives breast cancer metastasis (11). 

### 4.5 Association Between Breast Cancer Subtypes and TRAF4

#### 4.5.1 Molecular Subtypes of Tumours in Clinical Patients

Breast cancer is divided into four subtypes based on the biomarkers estrogen receptor (ER) and progesterone receptor (PR), and human epidermal growth factor receptor 2 (HER2). The four main subtypes of breast cancer include Luminal A (ER or PR positive, or both, HER2 negative, low proliferation), Luminal B (ER or PR positive, or both, HER2 negative, high proliferation), HER-2/neu type (HER2 positive and ER and PR negative), Basal cell-like type (HER2 negative, ER and PR negative; triple-negative breast cancer) (51).

TRAF4 nucleus staining was more pronounced in breast tumour specimens than normal breast tissue, and high TRAF4 nucleus expression was significantly associated with poorer overall survival in breast cancer patients. TRAF4 expression in ER-positive breast tumours was inversely correlated with the expression of p53-regulated pro-apoptotic genes, and SRC-3 combined with TRAF4 overexpression reduced the ability of p53 to induce apoptosis in ER-positive breast tumours (47). Tamoxifen significantly reduces the risk of cancer recurrence and metastasis in patients with estrogen receptor-positive breast cancer. Still, a proportion of patients treated with tamoxifen develop intrinsic or acquired resistance, and overexpression of TRAF4 leads to poor prognosis in patients with estrogen receptor-positive breast cancer treated with tamoxifen (52).

Patients with high TRAF4 expression have a poorer prognosis, and those with high TRAF4 expression combined with HER-2 positivity have an even worse prognosis (11). The co-expression of TRAF4 and Girdin proteins was significantly higher in HER-2 negative cases compared to HER-2 positive patients. Girdin is mainly expressed in the cytoplasm of breast cancer cells, but TRAF4 could facilitate its translocation from the cytoplasm to the nucleus (40).

There was a significant correlation between PRMT5 nucleus expression and HER-2-positive subtypes. TRAF4 is involved in the activation of the NF-κB signalling pathway by upregulating PRMT5 and promoting its nucleus expression, and together with HER-2 ectopic expression promoting NF-κB pathway activation, high PRMT5 combined with TRAF4 expression is a poor indicator of prognosis in breast cancer patients (41). TRAF4 is mainly expressed in the cytoplasm, and the cytoplasmic positivity rate is higher than the nucleus positivity rate. Nucleus expression of TRAF4 correlates with clinical stage, molecular/pathological staging and p53 status. Nucleus TRAF4 expression is higher in HER-2/Neu cells than in basal cell types, suggesting that TRAF4 in the nucleus may play an essential role in tumour growth and invasion and may help guide the choice of chemotherapy regimens. The expression of TRAF4 was higher in HER-2/Neu cells than in basal cells (53).

#### 4.5.2 Molecular Subtypes of Tumours in Cell Lines

MDA-MB-231 is a breast cancer cell line from patients with triple-negative breast cancer (TNBC) that is highly aggressive. MCF-7 is an estrogen receptor-positive subtype with weakly aggressive cells. TRAF2 and TRAF4 proteins were co-localised in two breast cancer cell lines, MCF-7 and MDA-MB231. In estrogen receptor-positive breast cancer cells (MCF-7), TRAF4 expression was higher in the nucleus than in estrogen receptor-negative breast cancer cells (MDA-MB231), with the opposite expression in the cytoplasm (42). TRAF4 was localized in the cytoplasm and nucleus of MCF-7 cells, and its expression was more robust in the nucleus than in the cytoplasm. And TRAF4 expression was higher in estrogen receptor-positive breast cancer cell lines than in estrogen receptor-negative breast cancer cell lines (45).

TRAF4 and β-catenin co-localize in the cytoplasm of MCF-7 and MDA-MB-231. TRAF4 promotes the proliferation of breast cancer cells by enhancing β-catenin expression (8). TRAF4 interacts with p70s6k in MCF7 and MDA-MB-231, leading to the proliferation of breast cancer cells (46). Wogonside inhibits breast tumor growth and metastasis by suppressing TRAF2 and TRAF4 expression *in situ* models of MDA-MB-231 cells (54). MTO1 binds to TRAF4 as a competitive endogenous RNA (ceRNA) in MCF-7 and MDA-MB-231, leading to decreased Ki-67 expression, inhibiting tumor activity, and reversing chemotherapeutic drug resistance (55). In estrogen receptor-positive breast cancer cells, TRAF4 binding to IRS-1 stimulates proliferation of breast cancer cell lines and attenuates sensitivity to chemotherapy (44).

#### 4.5.3 Molecular Subtypes of Tumours in the Genetic Level

High grade invasive ductal carcinoma (IDC) had significantly higher copy numbers of HER2 gene and TRAF4 gene than low/medium grade IDC, suggesting that TRAF4 and HER2 are synchronized in the progression of breast cancer infiltration (56). High levels of TRAF4 amplification were more common in invasive carcinoma and ductal carcinoma *in situ* (DCIS) than in columnar cell lesions (CCL) (57). Luminal A and B types are most common in male breast cancers, and increased TRAF4 gene copy number is present in male breast cancer patients with both, suggesting an association between TRAF4 and the molecular staging of male breast cancer (58).

Mediator subunit 1 (MED1) is a crucial ERa co-activator that plays a role in HER2-mediated tamoxifen resistance. MED1 gene amplification at the 17q12 locus correlates with TRAF4 gene amplification sites. It is associated with ER and HER2 positivity in breast cancer, triggering poor prognosis in breast cancer patients and leading to tumour resistance (59). Multiplex ligation-dependent probe amplification (MLPA) assays for infiltrating secretory carcinomas show increased copies of TRAF4 and HER2 genes, and HER2 with genes in the GRB7 (HER2 amplicon) 17q12-21 regions are vital discriminators. HER2+ clusters mediate higher proliferative activity, and TRAF4 may be associated with HER2+, leading to more aggressive tumours (60).

#### 4.5.4 Other Types of Classification

TRAF4 nucleus expression was significantly higher in non-invasive ductal carcinoma than in invasive ductal carcinoma, and TRAF4 nuclear expression was negatively correlated with breast cancer invasiveness. TRAF4 may promote breast cancer progression by altering the expression of cell nucleus (37). In epithelial breast cancer cells, TRAF4 depletion impairs EMT, while in mesenchymal-like cells, TRAF4 deficiency leads to loss of cell mobility. TRAF4-deficient cells exhibit reduced cell mobility (61). By studying tissue samples from 80 breast cancer patients (invasive ductal carcinoma, clinical-stage II-III) diagnosed by puncture biopsy, breast cancer patients with low TRAF4 expression benefited most from chemotherapy (50).

## 5 Regulatory Role in Other Tumors

### 5.1 Liver Cancer

TRAF4 is more highly expressed in hepatocellular carcinoma (HCC) cell lines and tissues than regular hepatocyte lines and adjacent non-cancerous tissues. Overexpression of TRAF4 in HCC tissues correlates with tumor number and vascular invasion. Induced by EMT through the PI3K/AKT signaling pathway activation, TRAF4 promotes HCC cell migration and invasion (62). High levels of TRAF4 promote intrahepatic cholangiocarcinoma (ICC) cell invasiveness by activating AKT signaling, and TRAF4 overexpression is associated with shorter overall survival and higher recurrence rates in ICC patients (63). The mRNA levels of TRAF4 were negatively correlated with miR-302c-3p expression. TRAF4 repair reverses the effect of miR-302c-3p on AKT-induced inhibition of EMT and HCC cell metastasis. MiR-302c-3p exerts tumor-suppressive effects in hepatocellular carcinoma by targeting TRAF4. Inhibition of the miR-302c-3p/TRAF4 axis may be a therapeutic target for hepatocellular carcinoma (64).

### 5.2 Lung Cancer

TRAF4 is a crucial molecule for AKT activation in lung cancer. TRAF4 reduces lung cancer glucose metabolism by inhibiting AKT pathway-mediated expression of Glut1 and HK2. TRAF4 is overexpressed in lung cancer and is essential for lung cancer cells to maintain tumorigenic properties such as glycolysis and xenograft tumor growth (10). TRAF4 is a target gene for miR- in small cell lung cancer (SCLC). ZFPM2-AS1, miR-3612, and TRAF4 constitute an SCLC competitive endogenous RNA (ceRNA) network. ZFPM2-AS1 is significantly upregulated in SCLC tissues and cells, and TRAF4 reverses ZFPM2-AS1 downregulation-mediated proliferation and invasion of SCLC cells *in vitro* and tumor function of growth (65). MiR-370 overexpression downregulated TRAF4 protein expression in non-small lung cancer (NSCLC) cells. Overexpression of TRAF4 reversed the growth inhibitory effect of miR-370 overexpression on NSCLC cells (66). TRAF4 stabilized the NOX complex by reducing NOX2 and NOX4-mediated lysosomal degradation. In turn, the NOX complex increases the level of endosomal ROS that can penetrate the cytoplasm, leading to NF-κB-mediated upregulation of ICAM1, which affects the tumor microenvironment and increases the invasiveness of cancer cells (67).

### 5.3 Endometrial Cancer

TRAF4 is upregulated in endometrial cancer (EC) tissues. TRAF4 overexpression decreases apoptosis and increases cell proliferation and migration. TRAF4 downregulation inhibits primary EC cells’ PI3K/AKT signaling pathway. Oct4 is a downstream factor of the PI3K/AKT pathway and positively regulates TRAF4. TRAF4 may increase cancer cell viability through the PI3K/AKT/Oct4 pathway to increase cancer cell viability. TRAF4 plays an oncogenic role in EC progression by regulating PI3K/AKT/Oct4 pathway (68). The decrease in TRAF4 decreased protein kinase B (AKT) phosphorylation, promoted DNA double-strand breaks, and reduced levels of DNA repair-related proteins, including phosphorylated DNA-dependent protein kinase (p-DNA-PK) and RAD51 recombinase (RAD51). The effect of TRAF4 on the sensitivity of endometrial cancer EC cells to oncologic agents such as olaparib was mainly mediated by AKT phosphorylation mediated. The sensitivity of EC to PARP1 inhibitors was enhanced by reducing TRAF4, and the combination of reduced TRAF4 expression and PARP1 inhibition reduced lethality in EC treatment (69).

### 5.4 Prostate Cancer

TRAF4 is a target gene of miR-519d-3p, down-regulated in prostate cancer cells. miR-519d-3p overexpression significantly reduced the expression of TRAF4 and its downstream TGF-β signaling pathway proteins in prostate cancer cells, thereby inhibiting the proliferation of prostate cancer cells (70). TRAF4 is an E3 ubiquitin ligase highly expressed in metastatic prostate cancer. It is a crucial player in the regulation of RTK-mediated prostate cancer metastasis. TrkA (a neurotrophin RTK) was identified as a TRAF4-targeted ubiquitination substrate that promotes cancer cell invasion, and inhibition of TrkA activity abrogates TRAF4-dependent cell invasion. TRAF4 promotes ubiquitination of K27 and K29 junctions in the TrkA kinase domain and increases their kinase activity. Mutations in TRAF4-targeted ubiquitination sites abolished TrkA tyrosine autophosphorylation and its interactions with downstream proteins. Reducing TRAF4 also inhibits nerve growth factor (NGF) stimulation of TrkA downstream p38 MAPK activation and invasion-associated gene expression. Elevated TRAF4 levels correlated significantly with increased NGF-stimulated invasion-related gene expression in prostate cancer patients, suggesting that this signaling axis is activated substantially during tumorigenesis. The results reveal a post-translational modification mechanism leading to aberrant non-mutant RTK activation in cancer cells (71). The tumor suppressor microRNA in miR-29a is one of the regulators of TRAF4 expression in metastatic prostate cancer, and the TRAF4 mRNA and protein expression is inversely correlated with miR-29a (72).

### 5.5 Esophageal Cancer

ZFPM2-AS1 is an oncogene for esophageal cancer (ESCC) cell growth and is regulated through upregulation of TRAF4 and activation of the NF-κB pathway. ZFPM2-AS1 is significantly upregulated in ESCC cells, and lowering ZFPM2-AS1 inhibits cell proliferation, migration, and invasion and promotes apoptosis in ESCC (73). TRAF4 is predominantly expressed in the ESCC cancer cells described in the cytoplasm at high expression, and TRAF4 overexpression is an independent risk factor for the overall prognosis of patients (74).

### 5.6 Colorectal Cancer

TRAF4 was overexpressed in colon cancer tissues and cells, and TRAF4 knockdown inhibited cell proliferation, invasion, and tumorigenesis *in vitro* and *in vivo* and induced apoptosis in colon cancer cells. SiRNA-TRAF4 significantly inhibited the expression levels of β-catenin, cyclinD1, and c-myc proteins in colon cancer cells. TRAF4 promoted the growth and invasion of colon cancer cells by enhancing the Wnt/β catenin pathway to promote colon cancer cell growth and invasion (75). TRAF4 catalyzes ubiquitination of CHK1 in several colorectal cancer (CRC) cell lines. Ubiquitination of CHK1 by TRAF4 at K132 after DNA damage is required for ATR-mediated CHK1 phosphorylation and activation. TRAF4 is highly expressed in chemotherapy-resistant CRC specimens and positively correlates with phosphorylated CHK1. Depletion of TRAF4 impairs CHK1 activity and sensitizes CRC cells to fluorouracil and other chemotherapeutic agents *in vitro* and *in vivo*. Activation of ATR-TRAF4-CHK1 signaling may lead to CRC chemotherapeutic drug resistance (76).

### 5.7 Glioma

TRAF4 is a direct target of miR-29a and a significant negative expression correlation between TRAF4 and miR-29a. MiR-29a is a crucial tumor suppressor in gliomagenesis by forming a negative feedback loop for TRAF4/AKT signaling, inhibits cell proliferation, migration, and invasion, and is an effective candidate for the treatment of glioma (77). MiR -29a/b/c induces apoptosis and inhibits glioma cell proliferation directly targeting TRAF4. MiR-29a/b/c promotes apoptosis in a p53-dependent manner through the TRAF4/AKT/MDM2 pathway, and miR-29a/b/c induces cell proliferation by blocking phosphorylation of AKT and GSK-3β and expression of cyclin D1. C-myc induces G1 arrest and inhibits tumor cell proliferation (78). Increased expression levels of DLEU1 and TRAF4 in glioblastoma multiforme (GBM) tissues promote GBM cell proliferation and regulate cell migration through the Hippo signaling pathway and Wnt signaling pathway. DLEU1 in GBM can act as a competitive endogenous RNA, thereby promoting GBM tumorigenesis (79).

### 5.8 Osteosarcoma

TRAF4 protein levels are significantly higher in osteosarcoma tissues than in normal bone tissue, and elevated TRAF4 causes tumor cell proliferation, promotes cancer cell colony formation, enhances osteosarcoma cell proliferation and invasion, and increases Ki67 expression through the AKT pathway (80). Decreased TRAF4 causes cell cycle arrest in the G1 phase. It promotes apoptosis through the action of TNF-α and nuclear factor κB, thereby affecting osteosarcoma cell proliferation, cell cycle, and apoptosis, providing a candidate molecular target for osteosarcoma prevention and treatment (81).

### 5.9 Ovarian Cancer

DR6 enhances the migratory ability of ovarian cancer cells. Reduced expression of DR6 inhibits the expression of MMP2 and MMP9 and increases the expression of E-cadherin. DR6 enhances the mobile capability of ovarian cancer cells through mitogen-activated protein kinase/ER and PI3K/AKT pathways. DR6, TRAF4, and KIF11 are upregulated in ovarian cancer, and DR6 may exert a significant oncogenic effect in ovarian malignancies through interaction with TRAF4 and KIF11 (82).

### 5.10 Squamous Cell Carcinoma of the Head and Neck

Nuclear expression of TRAF4 was observed in normal oral epithelium and highly and moderately differentiated squamous cell carcinoma of the head and neck (SCCN). In contrast, cytoplasmic expression of TRAF4 was observed in poorly differentiated SCCN, and the localization of TRAF4 was associated with the differentiation of SCCHN cells. TRAF4 is a common target of p53 family members, and p63 positively correlates with TRAF4 expression. P63, p73, and p53 transduce TRAF4 and promote tumor cell proliferation (83).

### 5.11 Role in Hematological Tumors

TRAF4 identification as a new direct target of miR-29s reveals that higher TRAF4 levels increase CLL response to CD40 activation and downstream nuclear factor-κB (NF-κB) signaling. In CLL, BCR inhibition of miR-29 expression *via* MYC allows for TRAF4 upregulation and more robust CD40-NF-κB signaling. This regulatory circuit can be disrupted by BCR inhibitors [Bruton tyrosine kinase (BTK) inhibitor ibrutinib or phosphatidylinositol 3-kinase (PI3K) inhibitor]. Mirna-dependent mechanisms can activate CD40 signaling/T-cell interactions in the CLL microenvironment, resulting in novel miR-29 regulated by BCR activity-TRAF4-CD40 signaling axis (84).

Multiple myeloma (MM) cells express CD40, and CD40L inhibits the growth and increases the apoptotic activity of MM cells by binding to Gp39. Gp39 treatment decreases TRAF4 expression, suggesting that CD40-dependent growth inhibition is associated with altered levels of TRAF4, which acts as an inhibitor of apoptosis in MM (85). Indole-3-hydroxy (I3C) and 3,3’-diindolylmethane (DIM) in vegetables may have a chemotherapeutic effect on T-cell acute lymphoblastic leukaemia (T-ALL) cells. In T-ALL, TRAF4 acts as an inhibitor of apoptosis in tumour cells. Treatment with DIM significantly reduced the expression of a transcript associated with human apoptosis (TRAF4) (86). Glucocorticoid-mediated E4BP4 promotes apoptotic effects in CEM lymphocytic leukaemia cells through upregulation, whereas TRAF4 expression in contrast to E4BP4 inhibits apoptosis in lymphocytic leukaemia cells (87) (**Table 2**).

**Table 2:** Tumour regulatory role of TRAF4.

Tumour Type	Signaling pathway	Major regulatory molecule	Functional linkage to TRAF4	Impact	PMID
Breast Cancer	NF-κB	PRMT5	Formation of zinc finger specific structure with TRAF4 (+)	Promote proliferation of breast cancer cells	25704480
	NF-κB	TRAF2	Enhance cytoplasmic expression of TRAF4 (+)	Promote cell proliferation and inhibit apoptosis	23743189
	NF-κB/AKT	RSK4	Reduce TRAF4 (-)	Inhibit proliferation, invasion and metastasis of breast cancer cells	29684350
	AKT	IRS-1	Reversal of cell growth arrest in TRAF4-silenced cells (+)	Increase proliferation of breast cancer cells	33991522
	β-catenin	–	Mediate transfer of β-catenin from the cytoplasm to the nucleus (+)	Promoting Wnt signaling pathway delivery in breast cancer	24990246
	β-catenin	SIAH1	Reduction in the number of S-phase cells, inhibition of apoptosis (+)	Reduce the growth inhibitory effects of chemotherapeutic agents	32671611
	TGF-β (cancer-promoting transforming growth factor-β)	SMAD	Effective migration, epithelial-mesenchymal transition (+)	Promotion of breast cancer metastasis	23973329
	TNF-α	–	Decrease in TRAF4 resulted in an increase in G1-phase cells and a reduction in S-phase cells (-)	Induction of apoptosis	24396457
	p70s6k/S6	p70s6k	Upward adjustment of TRAF4 (+)	Promote proliferation of breast cancer cells	25738361
	p53	SRC-3	Reduce upregulation of tumor suppressor p53 protein (+)	More resistant to apoptosis of breast cancer cells	23388826
	Rac1	Smurf1	Promote cell migration (+)	Migration of breast cancer cells	23760265
	–	MTO1	Inhibit TRAF4 activity (-)	Reversing chemotherapy resistance in breast cancer	30015883
Liver Cancer	PI3K/AKT signaling pathway	EMT	Increase tumor number and vascular invasion (+)	Promote migration and invasion of liver cancer cells	33349309
	AKT	–	Promote invasiveness of intrahepatic cholangiocarcinoma cells (+)	Resulting in shorter overall survival and higher recurrence rates	29749456
	AKT	miR-302c-3p	Inhibition of cell metastasis (-)	Tumor Inhibition	30087710
Lung Cancer	AKT	Glut1and HK2	Reduce lung cancer glucose metabolism (-)	Causing lung cancer cells to have reduced tumorigenic properties	24154876
	–	ZFPM2-AS1、miR-3612	Cell proliferation and invasion *in vitro* (+)	Tumor growth	32280300
	–	miR-370	Down-regulation of TRAF4 expression (-)	Inhibition of tumor growth	25976502
	NF-κB	NOX2 、NOX4 、sICAM1	Generation of endosomal ROS (+)	Promotion of tumor development	2882​​7764
Endometrial Cancer	PI3K/AKT/Oct4 signaling pathway	–	Increase cancer cell viability (+)	Increase proliferation and migration of cancer cells	30853613
	AKT	p-DNA-PK、RAD51	Increase TRAF4 and reduce the sensitivity of PARP1 inhibitors (+)	Increase lethality in EC treatment	32388810
Prostate Cancer	TGF-β signaling pathway	miR-519d-3p	Reduce TRAF4 expression (-)	Inhibit the proliferation of prostate cancer cells	29595452
	RTK	TrkA	Promotion of cancer cell invasion (+)	Activation of tumorigenesis	29715200
	–	miR-29a	Inversely proportional to TRAF4 expression (-)	Inhibition of metastatic prostate cancer	24100420
Oesophagal Cancer	NF-κB signaling pathway	ZFPM2-AS1	Upward adjustment of TRAF4 (+)	Promote cell proliferation, migration, and invasion	32065218
Colorectal Cancer	Wnt/β-catenin signaling pathway	β-catenin、cyclinD1 and c-myc protein	Promote growth and invasion of colon cancer cells (+)	Promote cell proliferation, aggression, and tumorigenesis, and inhibit apoptosis in colon cancer cells	25973026
	ATR-TRAF4-CHK1 signaling	CHK1	DNA damage (+)	Enhancement of chemotherapy resistance	32357935
Glioma	TRAF4/AKT signaling	miR-29a	Tumor suppressor (-)	Inhibit cell proliferation, migration, and invasion	30186853
	Phosphorylation of the TRAF4/AKT/MDM2 pathway, AKT, and GSK-3β	miR-29a/b/c	Induce G1 arrest and inhibit tumor cell proliferation (-)	Induce apoptosis and inhibit the proliferation of glioma cells	30348972
	Hippo signaling pathway and Wnt signaling pathway	DLEU1	Promote cell proliferation and migration (+)	Promote tumourigenesis	31257517
Osteosarcoma	AKT pathway	–	Tumour cell proliferation and promotion of colony formation (-)	Enhance tumor cell proliferation and invasion, increase Ki67 expression	25700355
	TNF-α、NF-κB	–	Promote proliferation and inhibit apoptosis in osteosarcoma cells (+)	Prevention and treatment of osteosarcoma affecting	25270078
Ovarian cancer	PI3K/AKT and mitogen-activated protein kinase/ER pathways	DR6	Enhance migration of ovarian cancer cells (+)	Carcinogenic effect	30186750
Squamous cell carcinoma of the head and neck	–	p63	p63 is positively correlated with TRAF4 expression (+)	Promote tumourigenesis	18087216
CLL	NF-κB	miR-29	miR-29 -TRAF4-CD40 signaling axis (-)	Cause poor prognosis of the tumour	33171493
**MM**	–	Gp39, CD40	Reducing TRAF4 expression (-)	Inhibit the growth of MM cells and increase apoptotic activity	10784400
**T-ALL**	–	I3C, DIM	Reducing TRAF4 expression (-)	Promote apoptosis of tumour cells	22514694
Lymphocytic leukaemia	–	E4BP4	Reducing TRAF4 expression (-)	Promote apoptosis in leukaemic cells	25101525

+ is positively correlated with the TRAF4 function; - is inversely correlated with the TRAF4 function.

## 6 Discussion

Thus, by summarising, we can clearly elucidate that TRAF4 affects the physiological and pathological functions of tumor cells in appealing tumors by participating in signaling pathways. Through the NF-κB signaling pathway, TRAF4 promotes cancer cell proliferation in breast cancer, oesophageal cancer, osteosarcoma, and chronic lymphocytic leukemia. Through AKT or PI3K/AKT signaling pathway, TRAF4 promotes cancer cell migration and invasion in breast, liver, lung, endometrial, osteosarcoma, glioma, and ovarian cancers. Through the Wnt signaling pathway, TRAF4 promotes cancer cell invasion in colorectal cancer and glioma. In addition, through TNF-α signaling, TRAF4 exerts anti-apoptotic effects on breast cancer and osteosarcoma. Through the β-catenin signaling pathway, TRAF4 increases resistance to chemotherapeutic agents in breast and colorectal cancers. Through the TGF-β signaling pathway, TRAF4 promotes cancer cell migration and invasion in breast and prostate cancers. In breast cancer, TRAF4 promotes cancer cell migration and invasion through the Rac1 signaling pathway. In prostate cancer, TRAF4 promotes cancer cell proliferation through the RTK signaling pathway. In glioma, TRAF4 promotes cancer cell proliferation and invasion *via* the Hippo signaling pathway.

**Figure 2:**
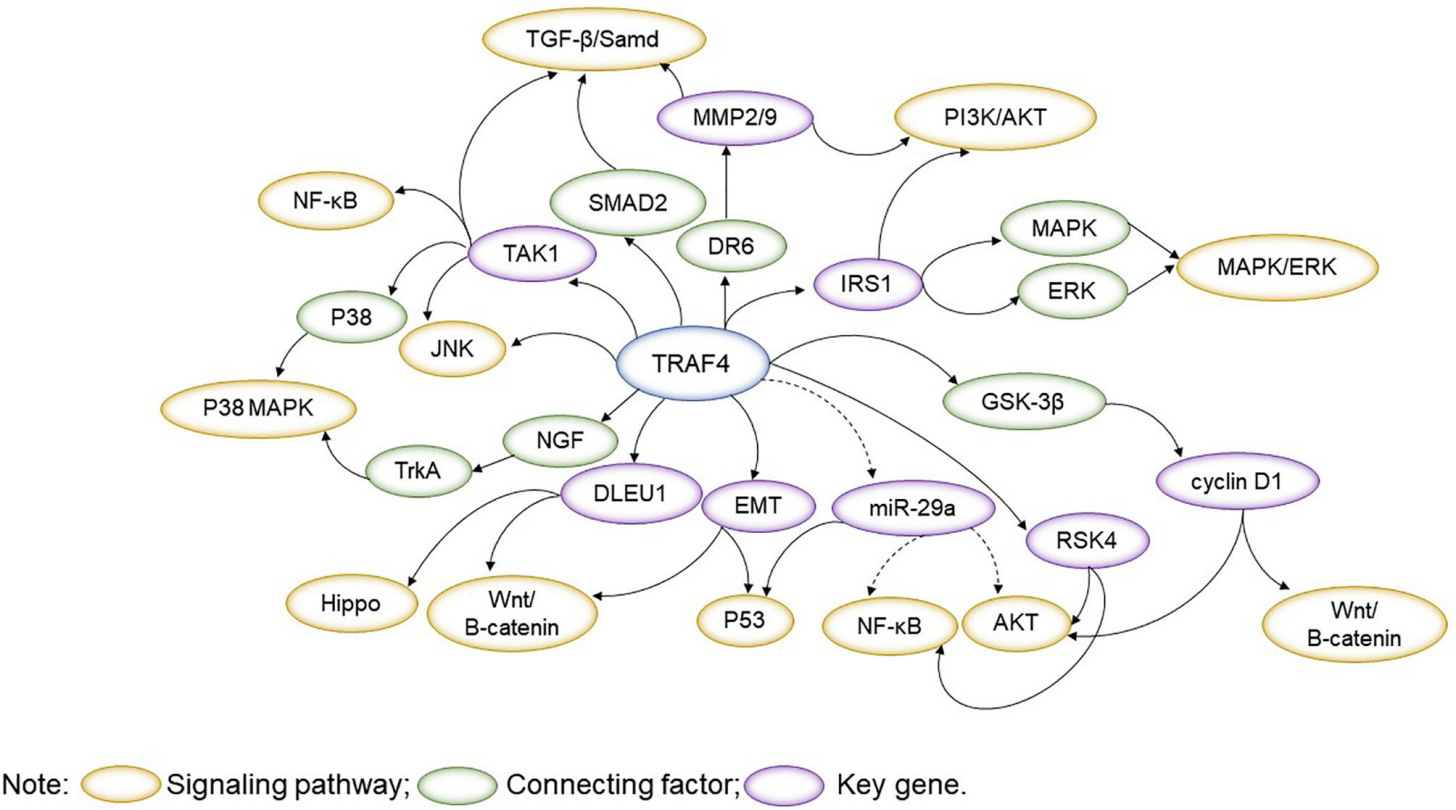
TRAF4 plays an essential role in physiological and pathological processes, which involves signalling pathways such as JNK, PI3K/AKT, MAPK/ERK, Wnt/β-catenin, TGF-β/Samd, NF-κB, Hippo. TRAF4-related signalling pathways are interlinked, with important regulators acting as bridges. By presenting the linkage of TRAF4-related signalling pathways throughout the text, it is easier to understand the regulatory role of TRAF4 in physiology and pathology.

TRAF4 plays an essential role in physiological and pathological processes, which involves signalling pathways such as JNK, PI3K/AKT, MAPK/ERK, Wnt/β-catenin, TGF-β/Samd, NF-κB, Hippo and others. Exploring the interconnections between signalling pathways with a focus on TRAF4 will help understand the role of TRAF4 as an interacting protein and provide a reference for the use of TRAF4 in disease (**Figure 2**).

TRAF4 promotes JNK activation by participating in the MAPK/ERK signaling pathway (9). TRAF4 binding to IRS-1 promotes its ubiquitination and phosphorylation, leading to activation of MAPK and PI3K/AKT signaling pathways. TRAF4 overexpression enhances IRS-1 tyrosine phosphorylation to encourage activation of ERK effector proteins (44). Increasing TRAF4 can promote NGF-stimulated activation of the p38 MAPK pathway downstream of TrkA and the expression of invasion-associated genes.

EMT induces TRAF4 to promote cancer cell migration and invasion by activating the Wnt/β catenin signaling pathway, and EMT induces p53 instability (16, 62). DR6 binding to TRAF4 enhances cancer cell mobility by enhancing MMP2 and MMP9 expression and participating in the PI3K/AKT pathway (82). TRAF4 involvement in the TGF-β/Samd signaling pathway can regulate MMP2, and MMP9 TRAF4 involvement in the TGF-β/Samd signaling pathway regulates the expression of MMP2 and MMP9 and induces P53 instability and tumorigenesis (16). Elevated TRAF4 expression regulates the TGF-β pathway and mediates an increase in phosphorylated SMAD2 and phosphorylated TAK1, triggering poor prognosis associated (11). In addition, TAK1 is involved in the NF-κB and JNK pathways and the p38 MAPK pathway (88).

Reducing TRAF4 can inhibit cancer cell proliferation, invasion and metastasis by downregulating the AKT signaling pathway, inhibiting the NF-κB pathway and engaging in RSK4 interactions (43). MiR-29a is an important tumor suppressor that promotes apoptosis and inhibits cell proliferation, migration and invasion by inhibiting the TRAF4/AKT/MDM2 pathway in a p53-dependent manner (77, 78). BCR promotes TRAF4 upregulation and activates the CD40-induced NF-κB signaling pathway through MYC inhibition of miR-29a expression (84). MiR-29a inhibits cell proliferation by blocking the phosphorylation of AKT and GSK-3β and cyclin D1 expression. High expression of TRAF4 significantly increased cyclinD1 and c-myc protein expression levels in cancer cells and activated the Wnt/β-catenin signaling pathway (75). DLEU1 can act as a competitive endogenous RNA that promotes elevated TRAF4 expression levels, promotes cancer cell proliferation through the Hippo signaling pathway and Wnt/β-catenin signaling pathway and regulate cell migration (79).

## 7 Conclusion

TRAF4 produces cell-specific and diverse biological effects in the regulation of cell differentiation, polarity, proliferation, and apoptosis, affecting embryonic development, rule of reactive oxygen species production, and mediating tumor formation and evolution. TRAF4 significantly enhances cancer development and progression in various malignancies, and its effects involve multiple signaling pathways such as AKT, NF-κB, Wnt, TGF-βand TNF-α. TRAF4 is a potential molecular target for cancer prevention and treatment, and the regulatory mechanism of TRAF4 needs to be further investigated. The study of TRAF4 can identify new targets suitable for tumor therapy and lay the foundation for developing new drugs and tumor drug resistance research.

## Author Contributions

XR wrote the review. RZ, RL, HZ, ZW, and CW discussed the review. All authors proofread the review. All authors contributed to the article and approved the submitted version.

## Funding

This work was supported by the National Natural Science Foundation of China (82070175), Natural Science Foundation of Hunan Province (2021JJ30937), Scientific program of Health Commission of Hunan Province (2022030442723), Changsha Municipal Natural Science Foundation (kq2014234).

## Conflict of Interest

The authors declare that the research was conducted in the absence of any commercial or financial relationships that could be construed as a potential conflict of interest.

## Publisher’s Note

All claims expressed in this article are solely those of the authors and do not necessarily represent those of their affiliated organizations, or those of the publisher, the editors and the reviewers. Any product that may be evaluated in this article, or claim that may be made by its manufacturer, is not guaranteed or endorsed by the publisher.
